# A Retrospective Weekly Rating of Perceived Exertion Measure May Assist Training Load Monitoring in Professional Tennis Players

**DOI:** 10.3390/sports14090385

**Published:** 2026-09-02

**Authors:** Diego H. Méndez, Federico J. Villalba, Santiago Soliño, Tim J. Gabbett

**Affiliations:** 1 Sports Physiotherapy Specialty, Favaloro University, Buenos Aires 1078, Argentina; federicojosevillalba@gmail.com (F.J.V.); lic.santiagosolino@gmail.com (S.S.); 2 Research Committee, KINÉ—Sports Clinic, Av. Córdoba 5779, Buenos Aires 1414, Argentina; 3 Department of Health, La Matanza National University, Buenos Aires 1754, Argentina; 4 Gabbett Performance Solutions, Sunshine Coast 4573, Australia; tim@gabbettperformance.com.au

**Keywords:** sports, athletes, concordance, internal load, session-RPE

## Abstract

The session rating of perceived exertion (s-RPE) is a validated tool for monitoring subjective training intensity across various sports. We aimed to assess the association between s-RPE recorded within 30 min post-training and a single retrospective weekly RPE (wRPE) reported at the end of the week. We tracked the training loads (TLs) of eight professional tennis players over one season and analyzed the association among three parameters: the weekly average of session-specific RPE (mRPE) vs. wRPE; weekly TL (s-RPE × duration) derived from both mRPE and wRPE; and week-to-week percentage changes in TL. We employed a nested model and a Bland–Altman analysis to evaluate association, bias, and limits of agreement. Our analysis revealed very large associations between mRPE and wRPE (r = 0.804) and between week-to-week TL progressions (r = 0.885). The association between TL measures themselves was nearly perfect (r = 0.974). Bland–Altman plots demonstrated superior agreement at lower loads, whereas higher loads were associated with greater variability. We conclude that a single self-reported measure at the end of the week was strongly associated with session-derived RPE, TL, and weekly TL progression in professional tennis players. Nevertheless, these two approaches are not interchangeable, and consistency in the chosen method is key for reliable TL monitoring in professional tennis players.

## 1. Introduction

Training load (TL) comprises both external load—representing the physical work performed (e.g., volume, distance)—and internal load, reflecting the athlete’s physiological, psychological, and biomechanical responses to that work [1]. Although appropriately prescribed TL results in positive adaptations, when these external and internal TLs are not adequately controlled, transient impairments in physical performance and substantial fatigue-related responses, including muscle damage and reductions in neuromuscular function, can occur [2]. In sports characterized by frequent competition, such as tennis, the repeated exposure to elevated TL may lead to the accumulation of fatigue across consecutive days, thereby increasing the risk of injury [3].

Monitoring TL in sports is a crucial practice both for coaches in prescribing training and for strength and conditioning coaches (S&C) or physiotherapists in preventing sports injuries and overtraining [4]. The association between spikes in TL progression more than absolute values, identified either through week-to-week changes [5,6] or using the acute–chronic workload ratio [7,8], and sports injuries has been a subject of debate in recent times [9,10]. Currently, there is no consensus on the degree of association between these measures [11,12], acknowledging that TL monitoring is part of a complex approach to injury prevention [13,14] but recognizing its limitations regarding injury prediction [15,16]. Nevertheless, only 50% of physiotherapists working with professional tennis players engage in TL monitoring [4]. Furthermore, TL monitoring should distinguish between physical conditioning and sport-specific practice, as these components serve fundamentally different purposes. Aggregating total TL without differentiating tennis-specific and fitness-related loads obscures the extent to which sport-specific exposure adequately prepares the athlete for the demands of competition [17].

With numerous objective and subjective TL monitoring modalities available in sports, the rating of perceived exertion (RPE) is one of the most widely used methods [18,19,20,21]. RPE has shown a significant association with endocrine responses (cortisol concentration and testosterone–cortisol ratio) [22], heart rate [23], mental effort [24], and blood lactate concentration [25] in tennis players, as well as in other sports [26]. In 1995, Foster et al. [27] adapted the RPE originally designed to assess the intensity of a specific part of training to evaluate the overall intensity of a complete session [28], naming it session RPE (s-RPE). This measure is multiplied by the duration of the stimulus to obtain a TL value for the session. The s-RPE was initially intended to be completed 30 min after exercise to assess the entirety of the session without the last part of the training disproportionately influencing the score [29]. However, subsequent studies have demonstrated that collecting these data both closer to [30,31,32,33] and up to 24 h after the end of the training [30,34] yields comparable values to those obtained at 30 min, demonstrating that s-RPE is a measure with high temporal robustness.

Key advantages of s-RPE over other methods for monitoring TL include its low economic cost, ease of collection, and, above all, the avoidance of performing maximum tests to calculate individual relative intensity. Specifically, in professional tennis, where it is common for monitoring personnel not to be present at all training sessions or international competitions, this method enables virtual data collection. Previous studies have demonstrated the feasibility of using technology for sports monitoring through questionnaires [35,36]. Additionally, the ease of virtual collection allows athletes to report data independently without the need for a team member to be present. However, this aspect can also be a drawback, as the responsibility for recording falls largely on the athlete, who may forget to complete the records or face obstacles in doing so daily [37], potentially leading to incomplete TL data. Collecting data weekly rather than after each session could reduce data loss while also decreasing the time burden on the athlete [38]. To date, only Phibbs et al. [34] have investigated the association between s-RPE values collected at 24 h and by recalling every single training session at the end of the week in adolescent rugby players, finding a high level of agreement but with a substantial typical error of the estimate, which may limit its practical applicability.

The association between different timings of TL recordings using the s-RPE method in tennis players is not well established. Therefore, the primary objective of this study was to assess the association between s-RPE recorded within 30 min post-training and the weekly RPE collected at the end of the training week and to evaluate their level of agreement using Bland–Altman analysis for monitoring TL in professional tennis players.

## 2. Materials and Methods

### 2.1. Design

We conducted a retrospective observational study in accordance with the STROBE (STrengthening the Reporting of OBservational studies in Epidemiology) guidelines and the ethical principles of the Declaration of Helsinki [39,40]. The Human Research Ethics Committee of ‘Hospital General de Agudos D. F. Santojanni’ (Ref. 14016) approved the data collection protocol. As part of a TL and wellness monitoring program at a specialized tennis physiotherapy institute, the principal investigator oversaw the players’ participation. Consequently, the players had familiarized themselves with the monitoring tools for at least 6 months prior to data collection and provided written informed consent for the use of their data. We monitored the cohort throughout an entire season (52 weeks); however, two players completed only 32 weeks due to injury or personal reasons.

### 2.2. Subjects

We enrolled a convenience sample of eight professional tennis players (6 males, 2 females; mean age 21.6 yr +/−2.3, range: 18–25). At the start of the season, participants held Association of Tennis Professionals (ATP) or Women’s Tennis Association (WTA) rankings between 43 and 1130. All participants competed in official international tournaments as their sole professional activity. Furthermore, we only included players who were available to train without physical restrictions.

### 2.3. Methodology

#### 2.3.1. Session Rating of Perceived Exertion

**Figure 1:**
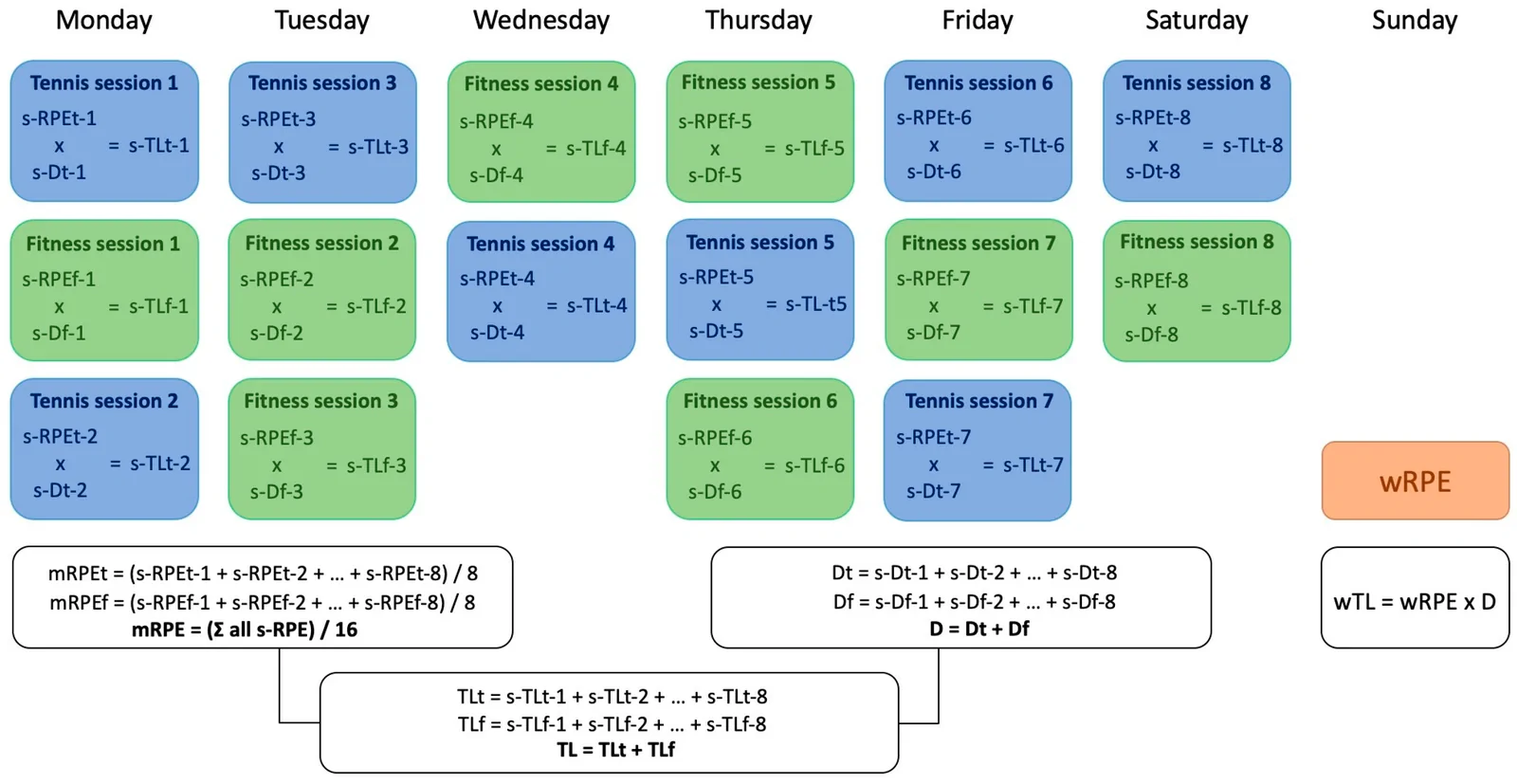
Example of the calculation of quantitative variables during a training week. The total number of sessions per week (represented here as *n* = 8 for tennis sessions and *n* = 8 for fitness sessions, totaling 16 sessions) is variable depending on the athlete’s schedule. The value of 8 sessions per discipline was selected arbitrarily for illustrative purposes only. s-RPE, session rating of perceived exertion; s-D, session duration; s-TL, session training load; wRPE, weekly rating of perceived exertion; mRPE, mean rating of perceived exertion. The “t” and “f” suffixes correspond to tennis and fitness, respectively.

Each player reported their average perceived exertion for every session using a modified Borg CR-10 scale [29]. Using a custom Google Forms^®^ (Google LLC, Mountain View, CA, USA) questionnaire on their smartphones, players also recorded the duration of each training session or competition (s-D; in minutes) within 30 min of completion [17]. We calculated the session training load (s-TL) by multiplying the s-RPE by the s-D (s-TL = s-RPE × s-D). We categorized training sessions into ‘tennis’ or ‘fitness’ based on the predominant load type, including both singles and doubles matches within the tennis category [17]. Finally, we monitored total load parameters and the specific categories (tennis and fitness) separately (Figure 1).

We calculated the weekly average s-RPE for both tennis (mRPEt) and fitness (mRPEf) by summing the s-RPE values for each category and dividing the total by the number of weekly sessions in that respective category. To determine the overall weekly average RPE (mRPE), we divided the sum of all s-RPE values by the total number of sessions performed that week, regardless of category. Additionally, we calculated the total weekly training duration (D) by summing the duration of every session (s-D) within the week, using duration as a proxy for external load. Similarly, we derived the total weekly training load (w-TL) by summing each s-TL within the same week. Finally, we categorized these total values into tennis and fitness for further analysis (Figure 1).

#### 2.3.2. Weekly Rating of Perceived Exertion

Beyond monitoring individual session loads, we administered a weekly questionnaire via Google Forms^®^ every Sunday night. Players had to complete this by 8 PM the following Monday. This tool allowed us to conduct wellness and injury surveillance while assessing the average perceived exertion for the entire week (wRPE). Specifically, players responded to the following prompt: ‘What was your average perceived exertion from last week during training/matches?’ To prevent recall bias, players could not access their previous s-RPE entries during the week. We calculated the weekly training load (wTL) by multiplying the wRPE by the total weekly duration (D) (wTL = wRPE × D). We used the sum of all daily post-session records for duration, as we did not collect a single retrospective duration measurement during the study. We did not categorize this value into tennis or fitness, as the wRPE represents the player’s holistic effort throughout the week.

#### 2.3.3. Weekly Progression of Training Load

We quantified TL progression by calculating the week-to-week percentage change for both weekly averages (TL) and weekly measures (wTL). We defined this metric as the ‘weekly progression percentage of TL’, which represents the relative fluctuations between consecutive weeks.

### 2.4. Statistical Analysis

We electronically transferred all data from Google Forms^®^ to Excel and conducted the analysis using R software (version 4.1.2, R Core Team, Vienna, Austria). We calculated descriptive statistics, reported as mean and standard deviation or median and interquartile range (IQR) based on data distribution, to detail the load variability for each player during fitness and tennis-specific sessions. To evaluate normality, we employed the Shapiro–Wilk test alongside visual inspections of histograms and box plots. Following the descriptive analysis, the presence of missing data was examined. Missing values accounted for 3% of all data cells; at the variable level, 3.3% of weekly records were missing for mRPE and 8.7% for wRPE. Given that the missing data mechanism was consistent with a missing at random assumption and that listwise deletion would have resulted in a 10.3% loss of cases, a single stochastic imputation was performed for these two variables using the missRanger package, a random forest-based algorithm selected for its ability to model non-linear relationships among mixed variable types without requiring a fully specified parametric imputation model [41]. The 20 weeks not recorded for the two players who withdrew before the end of the season were excluded from the dataset prior to analysis and were therefore not subject to imputation; missRanger was applied only to sporadic missing values within actively monitored weeks. The final dataset included 376 weekly observations across all eight players; when disaggregated into tennis- and fitness-specific records for the category-specific analyses, this yielded 724 observations.

We selected this approach because the nested structure was required to appropriately model the hierarchical dependence of sessions within weeks and players; deriving the correlation from the same model preserved methodological consistency without requiring a separate analytical procedure. To analyze the association between s-RPE and wRPE variables, we performed a nested model analysis. In this model, we treated ‘player’ and ‘training week’ as random variables and defined wRPE as the fixed variable to predict s-RPE [42,43]. We estimated the degree of correlation using the square root of the model’s *R*^2^. Specifically, we calculated the conditional *R^2^*, which accounts for the variance explained by the complete model, including both fixed and random effects:

$$R^{2}(C)=\frac{\sigma_{f}^{2}+\sigma_{\alpha }^{2}}{\sigma_{f}^{2}+\sigma_{\alpha }^{2}+\sigma_{t}^{2}}$$

Despite recording only a single overall RPE at the end of the week, we analyzed associations between wRPE and wTL across all mRPE and mTL variables. This allowed us to determine whether these weekly measures associated more strongly with total training load or the specific components of tennis and fitness training.

We employed a bootstrapping technique with 10,000 iterations, based on a normal distribution approximation, to estimate confidence intervals. To interpret the magnitude of correlations, we applied the following thresholds: r = 0.10–0.29 (small), 0.30–0.49 (moderate), 0.50–0.69 (large), 0.70–0.89 (very large), 0.90–0.99 (almost perfect), and 1 (perfect) [44]. Furthermore, we assessed the agreement between s-RPE and wRPE variables using Bland–Altman plots, displaying the mean bias and 95% limits of agreement alongside the differences between both measurement approaches [45]. Finally, we performed a stratified Bland–Altman analysis for TL by classifying observations into high- and low-load categories, using the median wTL as the cut-off point.

## 3. Results

We recorded a total of 4798 training sessions, with tennis-related activities accounting for 2606 (54.31%) of these entries. Table 1 summarizes the central tendency and dispersion values for self-reported exertion. The players completed a median weekly training duration of 983 min (range: 672–1181), with a median session duration of 68.57 min (IQR: 49.86–91.38). When categorized by stimulus type, fitness sessions yielded a median duration of 49.05 min (IQR: 41.22–60.00), whereas tennis sessions showed a significantly higher median duration of 89.03 min (IQR: 73.49–102.50).

We report the perceived exertion values for both measurement strategies, including weekly efforts and TL, in Table 2. Although we analyzed a robust dataset exceeding 700 individual data points, the small sample size of eight professional tennis players requires a cautious interpretation of these findings.

**Figure 2:**
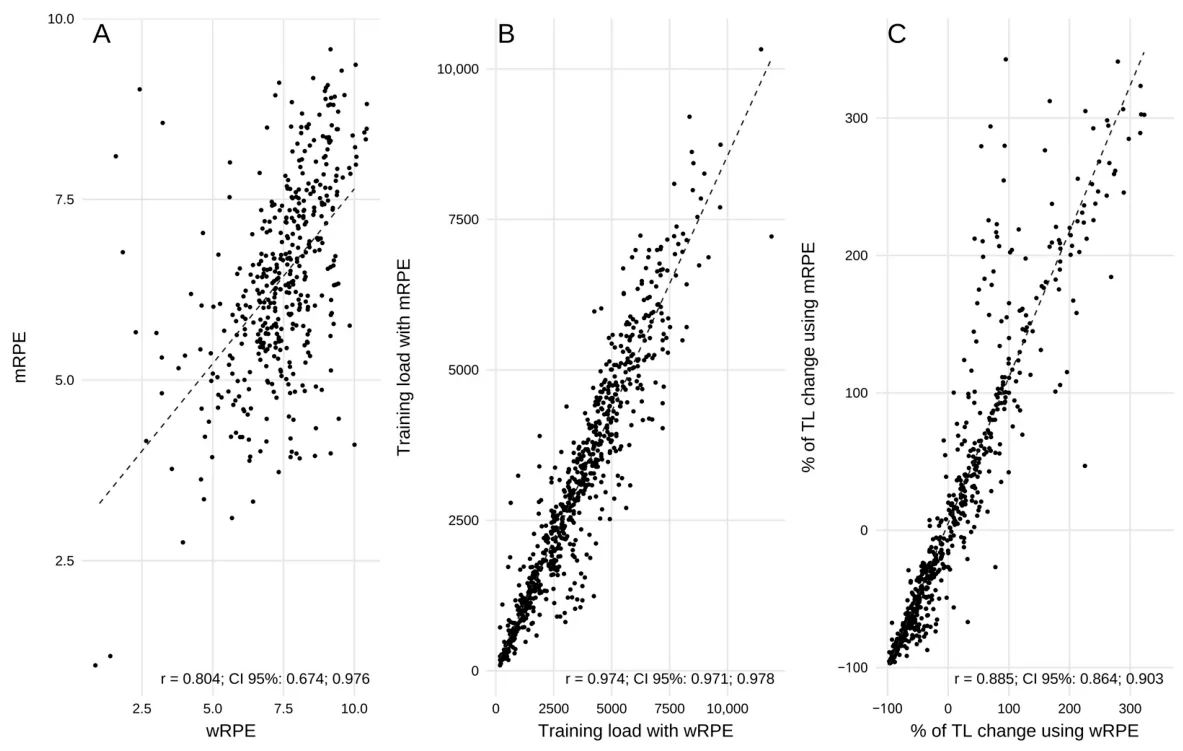
Scatterplot of training load measurements using (**A**) mean and weekly RPE, (**B**) mean and weekly training load, and (**C**) % of training load change using mean and weekly RPE. Correlations in this figure do not discriminate between tennis and fitness loads. wRPE, weekly RPE collected at the end of the week; mRPE, mean value of RPE measures during a week; TL, training load calculated with a measure of intensity (mRPE or wRPE) multiplied by the summation of the duration during a week; CI, confidence interval.

**Figure 3:**
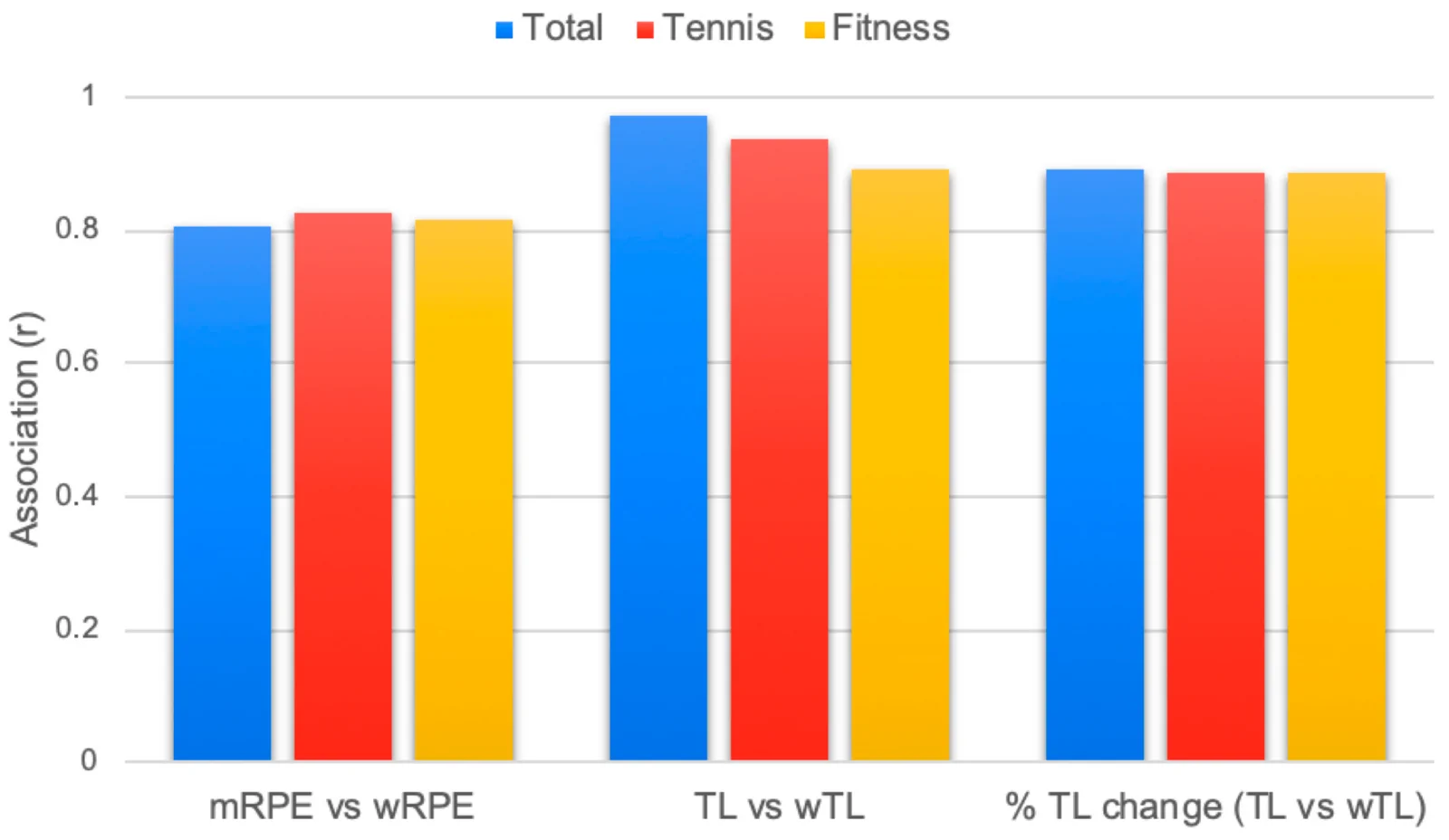
Associations between training load measurements using mRPE and wRPE, distinguishing between total loads, tennis loads, and fitness loads. wRPE, weekly RPE collected at the end of the week; mRPE, mean value of RPE measures during a week; TL, training load calculated with a measure of intensity (mRPE) multiplied by the summation of the duration during a week; wTL, training load calculated with a measure of intensity (wRPE) multiplied by the summation of the duration during a week.

Firstly, the nested model revealed a very large correlation between mRPE and wRPE (excluding duration) for total values (R^2^: 0.646; r = 0.804; 95% CI: 0.674; 0.976). We observed similar strong associations when analyzing individual categories, including tennis stimuli (mRPEt vs. wRPE, R^2^: 0.683; r = 0.827; 95% CI: 0.712; 0.942) and fitness stimuli (mRPEf vs. wRPE, R^2^: 0.745; r = 0.863; 95% CI: 0.653; 0.967) (Figure 2A and Figure 3).

Additionally, we used the nested model to estimate the correlation for TL (perceived effort multiplied by duration). This correlation was almost perfect for both the total value (TL vs. wTL, R^2^: 0.950; r = 0.974; 95% CI: 0.971; 0.978) and tennis load (TLt vs. wTL, R^2^: 0.901; r = 0.949; 95% CI: 0.925; 0.974), while remaining very large for fitness load (TLf vs. wTL, R^2^: 0.796; r = 0.892; 95% CI: 0.869; 0.912) (Figure 2B and Figure 3).

Furthermore, we evaluated the estimated correlation for the weekly progression percentage of TL using the nested model. We found large correlations for the total value (TL vs. wTL, R^2^: 0.783; r = 0.885; 95% CI: 0.864; 0.903), as well as for the specific progression percentages of tennis load (TLt vs. wTL, R^2^: 0.597; r = 0.772; 95% CI: 0.734; 0.803) and fitness load (TLf vs. wTL, R^2^: 0.783; r = 0.885; 95% CI: 0.866; 0.904) (Figure 2C and Figure 3).

**Figure 4:**
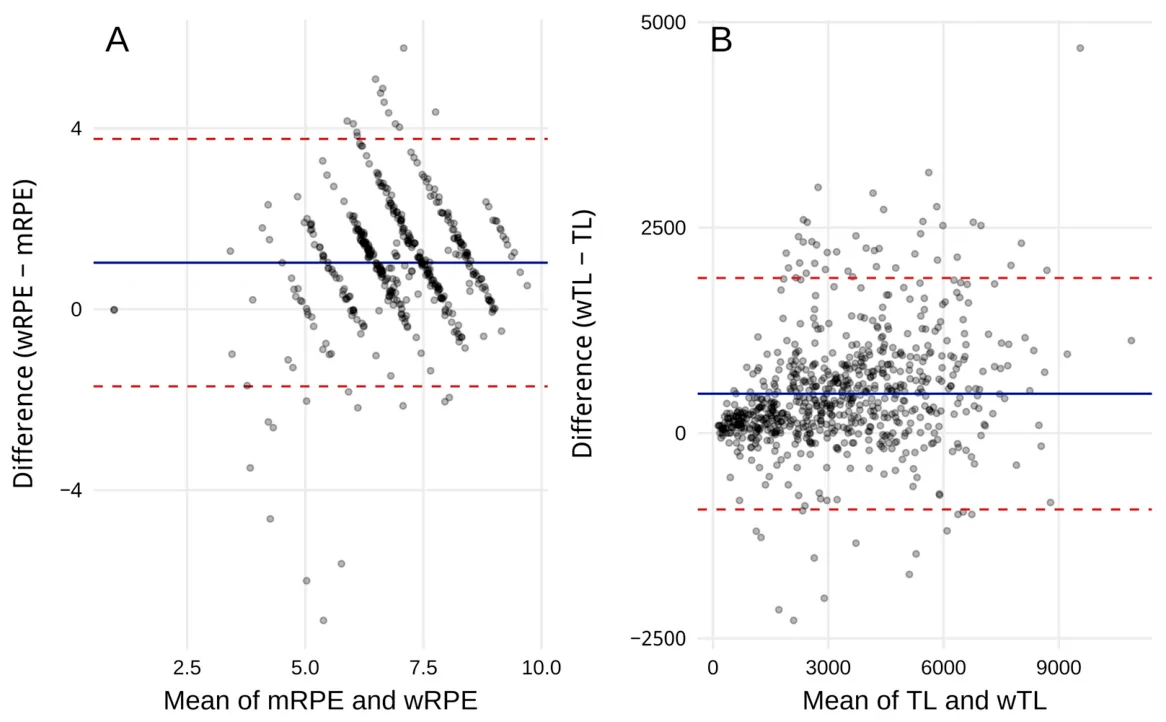
Bland–Altman analysis for (**A**) mean and weekly RPE, and (**B**) mean and weekly training load measures. wRPE, weekly RPE collected at the end of the week; mRPE, mean value of RPE measures during a week; TL, training load calculated with a measure of intensity (mRPE) multiplied by the summation of the duration during a week; wTL, training load calculated with a measure of intensity (wRPE) multiplied by the summation of the duration during a week.

**Figure 5:**
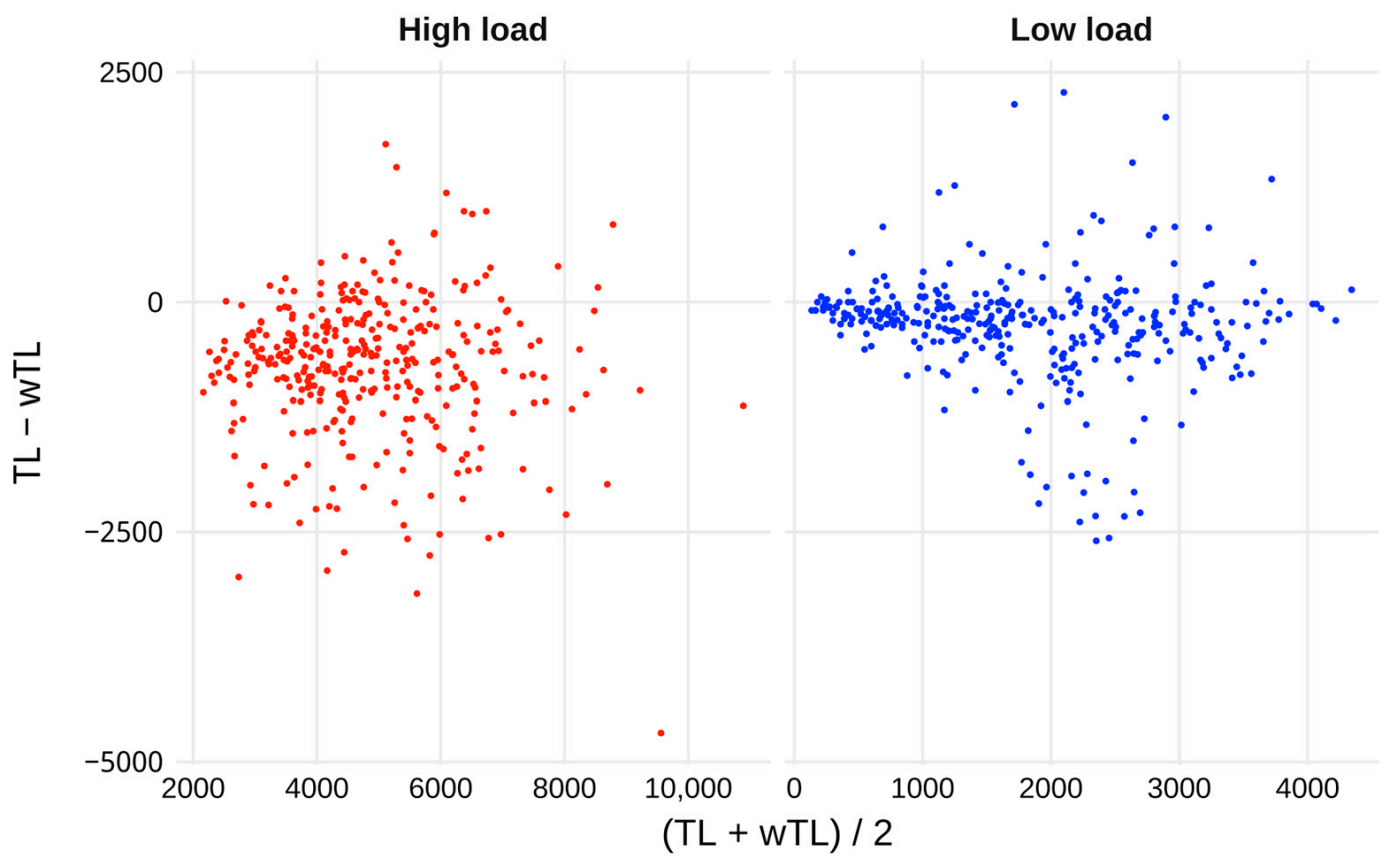
Stratified analyses of high and low training loads. TL, training load.

Bland–Altman analysis revealed a systematic positive bias for wRPE compared with mRPE. We observed wide limits of agreement and increasing dispersion at higher RPE values, suggesting limited agreement and a visual increase in variability at higher loads (Figure 4A). Conversely, the TL–wTL comparison demonstrated a small positive bias and narrower limits of agreement, suggesting superior agreement when including session duration (Figure 4B). Finally, our stratified analyses showed good agreement at low weekly loads, whereas higher loads were associated with greater variability and wider limits of agreement (Figure 5).

## 4. Discussion

To our knowledge, this study is the first to evaluate how different recording timelines for RPE are associated in professional tennis players. We investigated the degree of association among three key parameters for load monitoring. First, we compared the average internal load intensity recorded within 30 min of each session (s-RPE) against a single weekly retrospective measure (wRPE). Second, we repeated this analysis by incorporating session duration to assess total TL rather than intensity alone. Finally, we analyzed the correlation for week-to-week changes in TL between the two recording timings. Our findings revealed very large correlations for isolated RPE (r = 0.804–0.863) and for the weekly progression of TL (r = 0.772–0.885). Furthermore, we found almost perfect correlations for total TL (r = 0.974) and tennis-specific TL (r = 0.974). These results suggest that a single RPE measure reported at the end of the week is highly associated with the average effort, weekly TL, and weekly TL progression in professional tennis players.

Although we observed strong associations between RPE, TL, and weekly progressions across both measurements, the wide variability—particularly at higher intensity levels—suggests that wRPE represents more than a simple function of mean perceived intensity. Players reporting moderate mRPE often reported higher wRPE, potentially reflecting the influence of session frequency, duration, and the distribution of tennis versus fitness stimuli. These findings, however, underscore that a high correlation coefficient does not imply interchangeability between measures [46]. In support of this, our Bland–Altman analysis demonstrated limited agreement between mRPE and wRPE, characterized by a systematic positive bias and wide limits of agreement. Conversely, the TL–wTL comparison yielded narrower limits of agreement and reduced variability. This distinction highlights the necessity of incorporating session duration when estimating weekly internal load and supports the notion that exposure-related factors modulate the perception of weekly training load. Furthermore, our stratified analyses indicated that agreement between TL and wTL is load-dependent; we found superior agreement during low-load weeks, whereas high-load periods induced greater variability. These results emphasize that while incorporating duration improves overall agreement, researchers and practitioners should exercise caution when interpreting weekly training load estimates during periods of high accumulated load.

Although the difference between mRPE and wRPE in our study was statistically significant (1.01; *p* < 0.001), its practical relevance may be limited, even though the clinically meaningful change for this scale in professional tennis players remains unknown. Furthermore, this overestimation of perceived exertion at the end of the week—obtained through a single retrospective measurement compared to ratings taken 30 min following each session—may represent a holistic perception of the entire training week rather than simply the arithmetic mean of individual session RPE values. This may partly explain the systematic bias observed between methods.

Monitoring intensity via s-RPE immediately after sessions poses significant logistical challenges in tennis, as staff members responsible for data collection are rarely present at every session or international competition. Practitioners could partially address this issue by implementing fixed reporting times, such as the end of the day or week. Christen et al. [30] found that s-RPE recorded at 24 h did not differ significantly from 30 min recordings. Similarly, Scantlebury et al. [47] corroborated these findings, demonstrating correlations of 0.98 and 0.73 for s-RPE recorded at 24 and 72 h, respectively, when compared to 30 min measures. To date, only Phibbs et al. [34] have examined the association between end-of-week data and 30 min post-exercise recordings, reporting correlations of 0.88 for s-RPE and 0.87 for TL (s-RPE × duration) in adolescent rugby players. Although our results align with these values, we employed a different data collection methodology. While Phibbs et al. [34] required athletes to recall every individual session on Sunday, our tennis players provided a single, holistic weekly effort measure. We argue that Phibbs’ methodology would be impractical in tennis due to the high volume of weekly sessions characteristic of professional players [7,8,17]. Supporting this, our participants recorded TLs seven times higher than those of the adolescent rugby players in Phibbs’ study, a discrepancy we likely attribute to the older age and higher competitive level of our cohort.

We found that the weekly TL values recorded in our study (total load = 5856 AU; tennis load = 4074 AU) align with those reported by Méndez et al. [17] (total load = 6629 AU; tennis load = 4081 AU) in similar populations. These loads exceed the values reported by Moreno-Perez et al. [11] (5030 AU) and Myers et al. [12] (2880 AU), likely because those studies included junior players rather than professionals. Regarding self-reported intensity via the Borg CR-10 scale, our participants reported a median value of 6.5 for tennis sessions, which falls within the range (5–8) reported by Gomes et al. [23]. Furthermore, we observed a mean difference of 1.008 between the two intensity methods (s-RPE and wRPE). This result aligns with Foster et al. [48], who demonstrated that differences between measurement timings rarely exceed one RPE unit. However, despite the high correlations and the practical utility of these methods for monitoring TL, we argue against using them interchangeably due to these absolute value differences. Instead, practitioners should consistently implement a single method to ensure accuracy and longitudinal consistency in TL monitoring.

Implementing a single weekly measurement enhances the monitoring capacity of practitioners, particularly when overseeing professional tennis players remotely, with frequent time zone differences. This approach significantly reduces the time burden on players by eliminating the daily requirement for data entry. Simultaneously, it alleviates the workload for support teams by removing the need for daily oversight. Finally, this streamlined procedure optimizes data collection for TL research by minimizing data loss typically associated with inconsistent daily reporting.

Our study demonstrates significant practical utility for high-performance sports. However, we acknowledge several limitations. First, because our professional cohort exhibited training loads markedly different from those of youth athletes [11,12,34], we suggest that practitioners limit the application of this methodology to professional populations until further research confirms its generalizability to other age groups or performance levels. Second, we did not record weekly duration independently; instead, our TL and weekly progression calculations relied on aggregated daily duration data. This scenario limits the benefits of a low-burden weekly retrospective TL monitoring in the real world. Nevertheless, the strong association observed in intensity values provides a promising foundation for future studies to evaluate correlations using unique weekly duration entries. Furthermore, while our results support within-athlete consistency, we urge caution when interpreting the agreement or interchangeability between methods, as these findings may not extend beyond our specific sample. Finally, although the mixed-effects models appropriately account for the repeated-measures structure of the data, the effective inference remains constrained by the small number of independent participants (*n* = 8); the extended 52-week follow-up increases the density of observations per athlete but does not substitute for a larger and more heterogeneous sample. We also highlight that the elite status of these athletes inherently limits the available sample size, as is common when studying individual professionals competing at the international level. Our sample combined male and female players across a wide competitive range. Given the limited and unbalanced sample size, we did not include sex or ranking tier as covariates in the mixed-effects models, as this would have been statistically unstable. Differences in competition schedules, travel demands, and support staff availability across ranking tiers may influence exertion perception and recall accuracy. Future studies with larger and more balanced samples should explore these factors as potential moderators of agreement between s-RPE and wRPE. Additionally, the correlation coefficient derived from the conditional R^2^ is not directly equivalent to repeated-measures correlation or the intraclass correlation coefficient, which estimate within-subject association or absolute agreement, respectively. This metric should therefore be interpreted as a model-derived measure of association rather than a classical correlation coefficient.

## 5. Conclusions

When applied systematically, the weekly RPE method offers a practical, low-burden, and strongly associated alternative for monitoring training load and its progression in elite tennis environments. However, we recommend that practitioners and researchers implement one method consistently rather than switching between daily and weekly recordings, since these two monitoring approaches are not interchangeable due to the systematic bias and absolute differences observed.

## Figures and Tables

**Table 1:** Median data of s-RPE, wRPE, and number of weekly training sessions.

Measurement	Median	IQR
s-RPE	6.4	[5.5; 7.43]
s-RPEt	6.5	[5.75; 7.60]
s-RPEf	6.3	[5.21; 7.33]
wRPE	8.0	[7; 8]
Weekly training sessions	13	[9; 16]
Tennis training sessions	7	[5; 9]
Fitness training sessions	5	[3; 8]

RPE values are expressed in arbitrary units; training sessions are reported in number. IQR, interquartile range; s-RPE, session rating of perceived exertion; s-RPEt, s-RPE related only to tennis; s-RPEf, s-RPE related only to fitness; wRPE, weekly rating of perceived exertion.

**Table 2:** Average values and mean differences for mean RPE, weekly RPE, and training load measures.

Measurement	Weekly Metric (wRPE/wTL)	Session Metric (mRPE/TL)	Mean Difference (wRPE − mRPE)	Mean Difference (wRPE − mRPE) CI 95%	*p*-Value
mRPE	7.42 (1.46)	6.41 (1.36)	1.01 (1.37)	(0.88; 1.14)	<0.001
mRPEt	7.42 (1.46) *	6.59 (1.27)	0.83 (0.10)	(0.69; 0.97)	<0.001
mRPEf	7.42 (1.46) *	6.21 (1.61)	1.21 (0.12)	(1.05; 1.36)	<0.001
TL	6766 (3020)	5856 (2515)	910 (1269)	(782; 1037)	<0.001
TLt	4541 (2054)	4074 (1815)	467 (738)	(419; 567)	<0.001
TLf	2508 (1547)	2016 (1296)	493 (698)	(392; 542)	<0.001

Training load values are expressed in arbitrary units. Results are presented as mean (SD). wRPE, weekly RPE collected at the end of the week; mRPE, mean value of RPE measures during a week; letters “t” or “f” added to a word indicate that the values correspond specifically to tennis or fitness stimuli, respectively; CI, confidence interval. * Tennis and fitness categories do not change for wRPE because this measure does not discriminate between tennis and fitness.

## Data Availability

The data presented in this study are openly available in Mendeley Data at https://doi.org/10.17632/xhjt3fmggy.1 (accessed on 5 August 2026) [49].

## References

[1] Bourdon P.C., Cardinale M., Murray A., Gastin P., Kellmann M., Varley M.C., Gabbett T.J., Coutts A.J., Burgess D.J., Gregson W. (2017). Monitoring athlete training loads: Consensus statement. Int. J. Sports Physiol. Perform..

[2] Jones C.M., Griffiths P.C., Mellalieu S.D. (2017). Training Load and Fatigue Marker Associations with Injury and Illness: A Systematic Review of Longitudinal Studies. Sports Med..

[3] Halson S.L. (2014). Monitoring Training Load to Understand Fatigue in Athletes. Sports Med..

[4] Méndez D.H., Policastro P.O., De Oliveira Silva D. (2023). Injury Surveillance and Training Load Methods Used by Health Professionals in Tennis: An Online Multinational Survey. J. Sport Rehabil..

[5] Piggott B., Newton M., McGuigan M. (2009). The relationship between training load and incidence of injury and illness over a pre-season at an Australian Football League Club. J. Aust. Strength Cond..

[6] Gabbett T.J. (2016). The training-injury prevention paradox: Should athletes be training smarter and harder?. Br. J. Sports Med..

[7] Johansson F., Cools A., Gabbett T., Fernandez-Fernandez J., Skillgate E. (2022). Association between spikes in external training load and shoulder injuries in competitive adolescent tennis players: The SMASH cohort study. Sports Health Multidiscip. Approach.

[8] Johansson F., Gabbett T., Svedmark P., Skillgate E. (2022). External training load and the association with back pain in competitive adolescent tennis players: Results from the SMASH cohort study. Sports Health Multidiscip. Approach.

[9] Impellizzeri F.M., McCall A., Ward P., Bornn L., Coutts A.J. (2020). Training load and its role in injury prevention, part 2: Conceptual and methodologic pitfalls. J. Athl. Train..

[10] Impellizzeri F.M., Menaspà P., Coutts A.J., Kalkhoven J., Menaspà M.J. (2020). Training load and its role in injury prevention, Part I: Back to the future. J. Athl. Train..

[11] Moreno-Pérez V., Prieto J., Del Coso J., Lidó-Micó J.E., Fragoso M., Penalva F.J., Reid M., Pluim B.M. (2021). Association of acute and chronic workloads with injury risk in high-performance junior tennis players. Eur. J. Sport. Sci..

[12] Myers N.L., Aguilar K.V., Mexicano G., Farnsworth J.L., Knudson D., Kibler W.B.E.N. (2020). The Acute:Chronic Workload Ratio Is Associated with Injury in Junior Tennis Players. Med. Sci. Sports Exerc..

[13] West S.W., Clubb J., Torres-Ronda L., Howells D., Leng E., Vescovi J.D., Carmody S., Posthumus M., Dalen-Lorentsen T., Windt J. (2021). More than a Metric: How Training Load is Used in Elite Sport for Athlete Management. Int. J. Sports Med..

[14] Bittencourt N.F.N., Meeuwisse W.H., Mendonça L.D., Nettel-Aguirre A., Ocarino J.M., Fonseca S.T. (2016). Complex systems approach for sports injuries: Moving from risk factor identification to injury pattern recognition—Narrative review and new concept. Br. J. Sports Med..

[15] Dalen-Lorentsen T., Bjørneboe J., Clarsen B., Vagle M., Fagerland M.W., Andersen T.E. (2021). Does load management using the acute:chronic workload ratio prevent health problems? A cluster randomised trial of 482 elite youth footballers of both sexes. Br. J. Sports Med..

[16] Gabbett T.J. (2020). Debunking the myths about training load, injury and performance: Empirical evidence, hot topics and recommendations for practitioners. Br. J. Sports Med..

[17] Méndez D.H., Pierobón A., Gabbett T.J. (2022). Training Load Management in Professional Tennis Players During COVID-19 Lockdown: A Case Series Study. JOSPT Cases.

[18] Cousins B.E.W., Morris J.G., Sunderland C., Bennett A.M., Shahtahmassebi G., Cooper S.B. (2019). Match and Training Load Exposure and Time-Loss Incidence in Elite Rugby Union Players. Front. Physiol..

[19] Impellizzeri F.M., Rampinini E., Coutts A.J., Sassi A., Marcora S.M. (2004). Use of RPE-based training load in soccer. Med. Sci. Sports Exerc..

[20] Lupo C., Tessitore A., Gasperi L., Gomez M.A.R. (2017). Session-RPE for quantifying the load of different youth basketball training sessions. Biol. Sport..

[21] Mann R.H., Williams C.A., Clift B.C., Barker A.R. (2019). The validation of session rating of perceived exertion for quantifying internal training load in adolescent distance runners. Int. J. Sports Physiol. Perform..

[22] Gomes R.V., Moreira A., Lodo L., Nosaka K., Coutts A.J., Aoki M.S. (2013). Monitoring training loads, stress, immune-endocrine responses and performance in tennis players. Biol. Sport..

[23] Gomes R.V., Moreira A., Lodo L., Capitani C.D., Aoki M.S. (2015). Ecological validity of session RPE method for quantifying internal training load in tennis. Int. J. Sports Sci. Coach..

[24] Murphy A.P., Duffield R., Kellett A., Reid M. (2014). A descriptive analysis of internal and external loads for elite-level tennis drills. Int. J. Sports Physiol. Perform..

[25] Mendez-Villanueva A., Fernandez-Fernández J., Bishop D., Fernandez-Garcia B. (2010). Ratings of Perceived Exertion-Lactate Association During Actual Singles Tennis Match Play. J. Strength Cond. Res..

[26] Haddad M., Stylianides G., Djaoui L., Dellal A., Chamari K. (2017). Session-RPE method for training load monitoring: Validity, ecological usefulness, and influencing factors. Front. Neurosci..

[27] Foster C., Hector L.L., Welsh Mathew Schrager R., Green M.A., Snyder A.C. (1995). Effects of specific versus cross-training on running performance. Eur. J. Appl. Physiol. Occup. Physiol..

[28] Borg G. (1970). Perceived exertion as an indicator of somatic stress. Scand. J. Rehabil. Med..

[29] Foster C., Florhaug J.A., Franklin J., Gottschall L., Hrovatin L.A., Parker S., Doleshal P., Dodge C. (2001). A new approach to monitoring exercise training. J. Strength Cond. Res..

[30] Christen J., Foster C., Porcari J.P., Mikat R.P. (2016). Temporal robustness of the session rating of perceived exertion. Int. J. Sports Physiol. Perform..

[31] Fanchini M., Ghielmetti R., Coutts A.J., Schena F., Impellizzeri F.M. (2015). Effect of training-session intensity distribution on session rating of perceived exertion in soccer players. Int. J. Sports Physiol. Perform..

[32] Hornsby J.H., Green J.M., O’Neal E.K., Killen L.L., McIntosh J.R., Coates T.E. (2013). Influence of Terminal RPE on Session RPE. J. Strength Cond. Res..

[33] Uchida M.C., Teixeira L.F.M., Godoi V.J., Marchetti P.H., Conte M., Coutts A.J., Bacurau R.F.P. (2014). Does the Timing of Measurement Alter Session-RPE in Boxers?. J. Sports Sci. Med..

[34] Phibbs P.J., Roe G., Jones B., Read D.B., Weakley J., Darrall-Jones J., Till K. (2017). Validity of Daily and Weekly Self-Reported Training Load Measures in Adolescent Athletes. J. Strength Cond. Res..

[35] Clarsen B., Rønsen O., Myklebust G., Flørenes T.W., Bahr R. (2014). The Oslo sports trauma research center questionnaire on health problems: A new approach to prospective monitoring of illness and injury in elite athletes. Br. J. Sports Med..

[36] Ekegren C.L., Gabbe B.J., Finch C.F. (2014). Injury reporting via SMS text messaging in community sport. Inj. Prev..

[37] Shrier I., Raglin J.S., Levitan E.B., Mittleman M.A., Steele R.J., Powell J. (2014). Procedures for assessing psychological predictors of injuries in circus artists: A pilot prospective study. BMC Med. Res. Methodol..

[38] Saw A.E., Main L.C., Gastin P.B. (2015). Monitoring athletes through self-report: Factors influencing implementation. J. Sports Sci. Med..

[39] von Elm E., Altman D.G., Egger M., Pocock S.J., Gøtzsche P.C., Vandenbroucke J.P. (2007). The Strengthening the Reporting of Observational Studies in Epidemiology (STROBE) Statement: Guidelines for Reporting Observational Studies. PLoS Med..

[40] de Sanctis V., Soliman A.T., Daar S., Tzoulis P., Fiscina B., Kattamis C. (2022). Retrospective observational studies: Lights and shadows for medical writers. Acta Bio Medica Atenei Parm..

[41] Wright M.N., Ziegler A. (2017). ranger: A Fast Implementation of Random Forests for High Dimensional Data in C++ and R. J. Stat. Softw..

[42] Nakagawa S., Johnson P.C.D., Schielzeth H. (2017). The coefficient of determination R 2 and intra-class correlation coefficient from generalized linear mixed-effects models revisited and expanded. J. R. Soc. Interface.

[43] Bates D., Mächler M., Bolker B., Walker S. (2015). Fitting Linear Mixed-Effects Models Using lme4. J. Stat. Softw..

[44] Hopkins W.G., Marshall S.W., Batterham A.M., Hanin J. (2009). Progressive statistics for studies in sports medicine and exercise science. Med. Sci. Sports Exerc..

[45] Bland J.M., Altman D.G. (1986). Statistical methods for assessing agreement between two methods of clinical measurement. Lancet.

[46] Nevill A. (1996). Validity and measurement agreement in sports performance. J. Sports Sci..

[47] Scantlebury S., Till K., Sawczuk T., Phibbs P., Jones B. (2018). Validity of retrospective session rating of perceived exertion to quantify training load in youth athletes. J. Strength Cond. Res..

[48] Foster C., Boullosa D., McGuigan M., Fusco A., Cortis C., Arney B.E., Orton B., Dodge C., Jaime S., Radtke K. (2021). 25 years of session rating of perceived exertion: Historical perspective and development. Int. J. Sports Physiol. Perform..

[49] Méndez D.H. Weekly RPE in Tennis. Mendeley Data, V1 2025. https://data.mendeley.com/datasets/xhjt3fmggy/1.

